# “VelaMente?!” - Sailin in a Crew to Improve Self-Efficacy in People with Psychosocial Disabilities: A Randomized Controlled Trial

**DOI:** 10.2174/1745017901713010200

**Published:** 2017-11-13

**Authors:** Federica Sancassiani, Alessio Cocco, Giulia Cossu, Stefano Lorrai, Giuseppina Trincas, Francesca Floris, Gisa Mellino, Sergio Machado, Antonio Egidio Nardi, Elisabetta Pascolo Fabrici, Antonello Preti, Mauro Giovanni Carta

**Affiliations:** 1 Department of Medical Science and Public Health, University of Cagliari, Cagliari, Italy; 2 Center of Liaison Psychiatry and Psychosomatic, University Hospital, Cagliari, Italy; 3 Laboratory of Panic and Respiration, Institute of Psychiatry of Federal University of Rio de Janeiro (IPUB/UFRJ), Rio de Janeiro, (RJ), Brazil; 4 Physical Activity Neuroscience, Physical Activity Sciences Postgraduate Program - Salgado de Oliveira University, Niterói, Brazil; 5 Techniques for the Psychiatric Rehabilitation Degree Course, University of Trieste, Trieste, Italy

**Keywords:** Sailing, Sport, Psychosocial rehabilitation, Quality of life, Self-efficacy, Sense of coherence

## Abstract

**Introduction:**

It has been proposed that sailing can improve quality of life, personal and social skills of people with severe psychosocial disabilities. This study aimed to assess the efficacy of a psychosocial rehabilitative intervention focused on sailing on quality of life, self-efficacy and sense of coherence in people with severe psychosocial disabilities.

**Methods:**

The study was a randomized, with parallel groups, waiting-list controlled trial. Participants were 51 people with severe psychosocial disabilities. The intervention was a structured course to learn sailing in a crew lasting three months. A randomized group began the sailing course immediately after a pre-treatment assessment; the waitlist group began the sailing course after a three months period of treatments as usual. Participants were assessed before and after the sailing course, or the waiting list period, on the General Self-Efficacy scale (GSES), Sense Of Coherence scale (SOC) and Health Survey-short form (SF-12).

**Results:**

Self-efficacy significantly increased after the sailing course and decreased after treatment as usual (p=0.015). Sense of coherence and the levels of quality of life tended to improve after the sailing course, albeit below levels of statistical significance.

**Conclusion:**

When compared to more traditional psychosocial rehabilitative activities, an intervention focused on sailing in a crew positively impacts the sense of coherence and the levels of quality of life and significantly improves self-efficacy of people with severe psychosocial disabilities. Further longitudinal research is required.

## 1 INTRODUCTION

People with psychosocial disabilities usually report poor quality of life (QoL) [1, 2] and difficulties in social functioning [3, 4]. QoL refers to the perceived overall wellbeing, including physical, psychological and social functioning [5]. Several psychosocial factors can be related with the perceived QoL such as self-efficacy [6, 7], also in people with severe psychosocial disabilities [8, 9].

Self-efficacy is widely defined as the perceived ability to perform a task, take control of an action and/or achieve a desired goal [10-12]. It is a key concept in the theories about motivation [13-15] and healthy behaviors promotion [16, 17], such as physical activity [18], also in people with psychosocial disabilities [19]. Self-efficacy can be considered both as an outcome of physical activity programs, which may increase the perception of being in control of actions, and a determinant of adherence in attending them, since people with higher self-efficacy are more likely to pursue goal-oriented programs [20, 21].

Beside self-efficacy, wellbeing is related to a strong sense of coherence (SoC) and a positive perceived QoL [9, 22, 23]. SoC concerns the ability to understand, manage and give meanings to life events, in order to maintain good health and wellbeing [23]. A strong SoC is associated with higher levels of moderate to vigorous physical activity in young adolescent [24] and improves in people with severe psychosocial disabilities attending structured activities, such as a sport, in the form of lifestyle intervention program [25]. SoC is associated with QoL and perceived health in both Western and non-Western countries [22]. Moreover, SoC is strongly related to perceived health, especially mental health [23], so it is important to take it into account evaluating the impact of a treatment on QoL in people with psychosocial disabilities.

Interventions aimed at improving self-efficacy and SoC might have important effects in people with psychosocial disabilities well beyond their impact on QoL and wellbeing. Psychosocial disabilities cause an elevated burden on the affected people in terms of lost opportunities. Self-perceived stigma further increases the sense of inefficacy because of lost opportunities that is caused by psychosocial disabilities. When people are offered a chance of employing their time in activities from which they are often kept apart, they benefit from the opportunity [26]. In addition, the stigma attached to psychosocial disabilities makes worse the perceived QoL of people affected more than in other diseases [27]. In particular, the belief that not only individuals with a psychosocial disability are incurable, but also that their disability is irreversible, is deeply rooted both in public opinion and among health workers [28]. The bio-psychosocial model of disability proposed by the the World Health Organisation in the International Classification of Functioning Disability and Health – ICF [29] outlines that disability is a complex phenomenon, reflecting an interaction between individual characteristics, also about the body, and features of the society in which the individual lives. Hence, many aspects of disability are socially and environmentally determined issues and not all attributes of an individual. This model states that the community has the responsibility to recognize disability and provide supports for people with disabilities, in order to facilitate their participation as equal and valued citizens.

It might be advanced that a rehabilitative program, able to increase self-efficacy and SoC of the participants, might improve their QoL and wellbeing, as well as might reduce self-perceived stigma, one of the mechanisms that could contribute to the persistence of stigma in the society [30].

Sport activities and exercise are often used to implement rehabilitative programs for people with psychosocial disabilities, since they join an entertainment function with socialization goals and other, more specific aims [31]. Within this perspective, sport activities might exert a strong antidote against stigma [26].

In our culture, sport activities are socially appreciated and they are considered as a powerful vehicle to promote social relations, and physical and psychological wellbeing. Furthermore, people with severe psychosocial disabilities often reported higher levels of sedentary behavior during waking hours [32]. Several systematic reviews and meta-analyses point out that physical activity and sports can be proposed as a potential treatment to improve mood in people with depression [32-38], as well as health outcomes [39] and QoL [40] in people with schizophrenia.

Indeed, sport activities have often been proposed as rehabilitative interventions for people with severe psychosocial disabilities [39, 40]. Recently, a randomized controlled trial (RCT) [41, 42] evaluated the efficacy of a rehabilitative intervention focused on sailing on several clinical and psychosocial outcomes of people with severe psychosocial disabilities. Based on recovery promotion [43], this study pointed out the efficacy of sailing to improve the quality of life, social skills and psychopathological residual symptoms of participating patients [41, 42].

Within this perspective, and to the goal of adapting sailing to the needs of people with psychosocial disabilities [43-48], the project “VelaMente?!” was established as a psychosocial rehabilitation intervention for people with severe psychosocial disabilities. In Italian, the word “VelaMente?!” is a pun made up by joining the words “Sailing” (Italian: “Vela”) and “Mind” (Italian: “Mente”), which literally resembles the adverbs “Really?!” or “Indeed?!”. The project “VelaMente?!” focuses on a structured course to learn sailing in a crew.

This project was proposed in order to confirm and extend findings of the already mentioned RCT [41, 42] by recruiting a new sample and providing other changes: the enrollment of a wider sample of people with severe psychosocial disabilities (N 64 instead of N 40); a more structured sailing course, including theoretical and practical lessons overall similar to a basic sailing course, but adapted to the needs of each participant; the concurrent employment of sailing teachers and mental health operators; the choose of two regular sailboats usually used by crews of sailors during regattas.

The present paper refers to the following aims of the project “VelaMente?!”:

1. to confirm the hypothesis that sailing in a crew improves the QoL of people with severe psychosocial disabilities, as demonstrated in the pilot study [41];
2. to verify whether sailing in a crew can improve the SoC and if this improvement is related with perceived QoL.
3. to verify whether sailing can improve the self-efficacy and whether this improvement is related to perceived QoL.

## 2 METHODS

### 2.1 Design

**Fig. (1):**
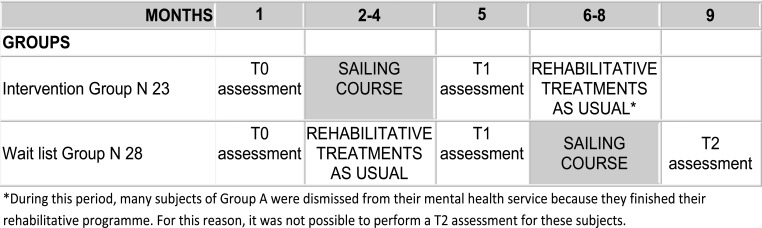
Study design.

The study was a randomized waitlist-controlled trial with parallel groups, with the experimental intervention period, lasting three months, crossed between two groups (Fig. **1**).

#### 2.1.1 Sample and Inclusion/Exclusion Criteria

A pool of 64 subjects was recruited among users of two public mental health services in Sardinia, Italy; one half of participants were from a residential mental health service of the Department of Mental Health of Carbonia-Iglesias and the others from the Center of Liaison Psychiatry and Psychosomatic, University Hospital of Cagliari.

The inclusion criteria were: 1) Schizophrenia, Affective Psychosis, and/or Personality Disorder diagnosis according to ICD-10 criteria [49]; 2) aged ≥ 18 years old; 3) being a user of the mental health service for two years at least; 4) signing the informed consent; 5) presenting a medical note for no agonistic sport activity.

The exclusion criteria were: 1) a strong medical contraindication for sport activities; 2) not being in clinical remission, with episodes of psychotic episode in the last three months.

**Fig. (2):**
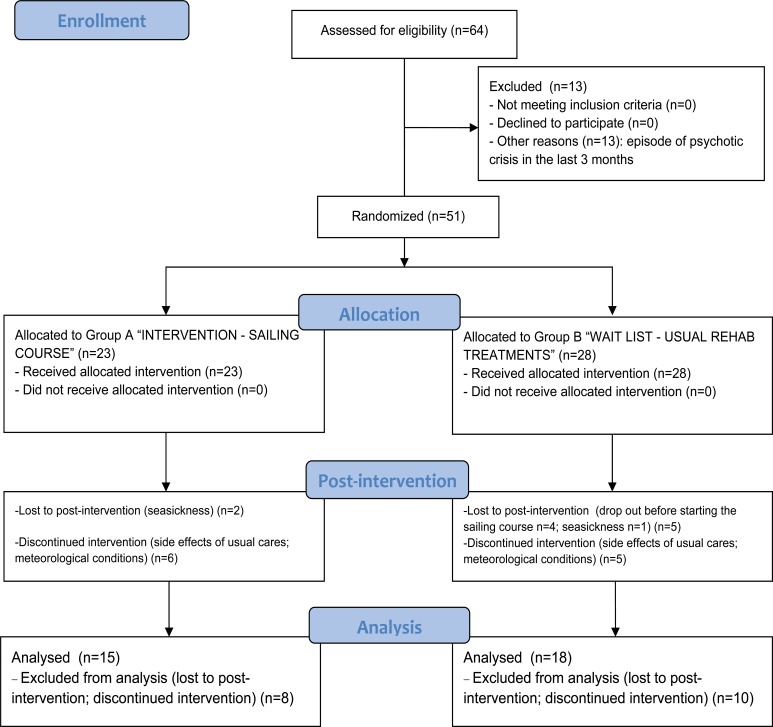
CONSORT flow diagram.

Among the starting pool, 13 (20%) users were excluded because they suffered from episodes of psychotic episode in the last three months (Fig. **2**).

#### 2.1.2 Randomization

At the beginning of the trial, each subject was randomized by a computer generated list, regardless of their mental health service. Expecting a higher percentage of dropout in the waiting list group, subjects were assigned to the treatment (Group A) and to the wait list (Group B) as following:

Group A: N 23 subjects attending the experimental intervention (three months sailing course) plus drugs treatments as usual. At the end of the sailing course (experimental intervention), they came back to the usual rehabilitative activities offered by their mental health service plus drug treatment as usual;

Group B: N 28 subjects (waitlist) attending the usual rehabilitative activities in their mental health service plus drug treatment as usual (three months). Then, when the Group A completed the sailing course, they started with the experimental intervention (three months sailing course) plus drugs treatments as usual. At the end of the sailing course, they started again with the usual treatments (See Figs. **1** and **2**).

#### 2.1.3 Intervention

The experimental treatment was a psychosocial rehabilitative intervention based on learning to sail in a crew by attending a structured course. The course was conducted in a sailing school sited in a touristic harbor in the eastern part of the Gulf of Cagliari (Italy). Participants reached the harbor from their two mental health services sited in Iglesias (distant almost 75 Km) or Cagliari (distant almost 20 Km) by a caravan driven by a mental health operator.

The course lasted three months, two lesson/week. Each lesson lasted almost four hours. The entire course consisted in a theoretical module of four lessons about principle of sailing (*i.e.*: winds and the Wind Rose; components and tools into the sailboat; knots; roles and group dynamics into a sailing crew; sails typology; maneuvers in the harbor and in the open sea; safety in the sailboat; *etc*…) and in a practical module of eight lessons in the open sea or in the harbor, in which the participants could apply this knowledge. The lessons took part directly on sailboats, in the touristic harbor and in the contiguous open sea, also on the bases of weather conditions, or in a dedicate room of the school. Each lesson involved a group of 8-10 participants, two regular sailboats usually used by crews of sailors for regattas, two skippers, two psychologists and two trainees attending the “Techniques for the psychiatric rehabilitation” Degree Course of the University of Cagliari, Italy. The participants were steadily motivated to enhance their learning ability and encouraged to discuss their impressions and feelings about the experience among the group or individually, in order to improve their social skills. Finally, it was spontaneously promoted the typical relaxed climate of sailing crews and environment.

The rehabilitative treatment as usual (rTAU) consisted in more traditional psychosocial rehabilitation activities usually conducted in the mental health services, such as self-help groups, group-supported work in the garden, group-laboratories of art therapy. Even if they were not evidence-based psychosocial rehabilitation activities, they shared with the experimental intervention some features that allowed a comparison, such as the group setting, the possibility to socialize and to stay in outdoor places, the continuity of the sessions.

#### 2.1.4 Assessment

The evaluators (N = 4) were blinded to the allocation of the subjects to the groups. However, since patients often talked about their sailing experience, blindness was difficult to preserve. Evaluators (two psychiatrists and two psychologists) were trained to administer the assessment instruments, and had four years professional experience at least with patients with severe psychosocial disabilities. The assessment took place in dedicate rooms into the mental health services involved in the project.

As shown in Fig. (**1**), for the intervention group the assessment points were: pre-treatment (T0) and post-treatment evaluation (T1), before and after the sailing course; for the wait list group, the assessment points were: pre- (T0) and post- (T1) waiting list period (treatments as usual), and post-treatment (T2) at the end of sailing course. It was not possible to assess again the Group A 3 months after the post-treatment evaluation (T2) because many subjects were dismissed from their mental health service due to the end of their rehabilitative programme.

#### 2.1.5 Instruments

- The 13-item *Sense of Coherence Scale* (SOC) [50] in the Italian version [51] was used to measure sense of coherence. The SOC-13 is a self-report questionnaire consisting of three components: comprehensibility, manageability, and meaningfulness that are equally weighted. The items were rated on a 7 point Likert scale and the total score ranges from 13 to 91, with higher scores indicating a higher SOC.
- The *Short Form Health Survey (SF-12)* [52] in the Italian version [53] was used to measure quality of life (QOL). The SF-12 consists of two domains: mental and physical health. The items were rated by 3 or 5 points Likert scale. The total score ranges from 12 to 47, with higher scores indicating a higher QOL.
- The *General Self Efficacy Scale (GSES)* [54] in the Italian adaptation [55] was used to measure self-efficacy. The GSES is a 10 item self-report questionnaire rated by 4 points Likert scale. The total score ranges from 10 to 40, with higher scores indicating a higher self-efficacy.
- An *ad hoc* report was used to collect the socio-demographic data, clinical status and information about usual cares (see Table **1** for details).

#### 2.1.6 Statistical Analysis

The data refer to the comparison between the subjects attending the sailing course (Group A + Group B: experimental treatment) and the subject in the wait list (Group B: usual treatments).

As shown in the Fig. (**2**), those subjects that skipped more than more than four lessons were excluded from the analyses (n = 18, because lost to post-intervention or discontinued intervention), in order to better assess the effectiveness of the experimental intervention.

Data were analyzed with the Statistical Package for Social Science (SPSS) for Windows (Chicago, Illinois60606, USA), version 21.

The descriptive statistics (mean ± sd; %) were used for nominal and continuous variables to point out the characteristics of the samples (age; gender; marital status; education; diagnosis; psychosocial variables).

A series of Chi-Square (nominal variables) and one-way ANOVA (continuous variables) were used to verify the homogeneity between the two groups (“experimental treatment” - “rehabilitative treatment as usual”) at T0, about all variables.

A series of repeated measures ANOVA were used to assess the expected differences “time*group” for the dependent variables (“sense of coherence”; “quality of life”; “self-efficacy”).

## 3 RESULTS

### 3.1 Socio-Demographic, Clinical and Psychosocial Characteristics of the Samples

Socio-demographic, clinical and psychosocial features of the study samples at T0 are shown in Table **1**.

Any statistically significant differences regarding gender, age, marital status, education, diagnosis, levels of sense of coherence, self-efficacy and quality of life were found between the “experimental treatment” (sailing course) group to the “rehabilitative treatment as usual” (wait list) group at T0.

#### 3.1.1 Drop-Out and Discontinuity During the Sailing Course Attendance

As shown in Fig. (**2**), two subjects in the “experimental treatment” (sailing) group dropped out at post-treatment because seasickness and other six attended the sailing course discontinuously (more than four lacked lessons) because some sides effects of drug therapy or weather conditions that required some adjustments in the lessons program. In the “rehabilitative treatment as usual” (wait list) group, five subjects dropped out (one because seasickness; four before starting the sailing course) and five attended the sailing course discontinuously (more than four lacked lessons) because some sides effects of drug therapy or meteorological conditions that required some adjustments in the lesson program.

At T0, there were any statistical differences about mean scores on GSES, SOC and SF-12 between dropped-out subjects and those included in data analyses.

#### 3.1.2 Outcome Measures

As shown in Table **2**, there were statistically significant differences “time X group” on self-efficacy (GSES: p = 0.015), with a clear improvement for the sailing group and a decreasing trend in the wait list group.

It was not found any statistically significant difference “time X group” regarding sense of coherence (SOC: p = 0.357) and quality of life (SF-12: p = 0.627). However, it was observed a very low improvement trend expressed by mean scores in the sailing group and a relative stability in the wait list group on both outcomes.

## 4 DISCUSSION

This paper focuses on the results of the project “VelaMente?!”, a psychosocial rehabilitative intervention with a cross-over RCT design, which was based on a structured sailing course in a crew lasting three months and involving 51 people with severe psychosocial disabilities.

The results concern three well-being outcomes: self-efficacy, sense of coherence and quality of life, and the study tested the hypothesis that they were liable to improvement by a structured physical activity [19, 21, 24, 25]. The subjects attending the sailing course showed a statistically significant improvement in their levels of self-efficacy, when compared to the subjects in the waiting list group attending more traditional group psychosocial rehabilitative interventions such as self-help, gardening or art therapy in the waiting list group. Moreover, in the waiting list group self-efficacy tended to decrease.

A recent pilot RCT [56] about the effectiveness of a psychodynamic group art therapy for people with severe psychosocial disabilities shown similar findings about self-efficacy: it improved in a statistically significant way more in the “treatment as usual” group (a mix of individual and/or group cognitive-behavioral or modify psychodynamic interventions; occupational therapy, music therapy, cognitive and social skill training, excursions, relaxation and sport) than in the “art therapy” group. Hence, self-efficacy seems to be more susceptible of improvement in people with severe psychosocial disabilities after a rehabilitative intervention including structured group activities, delivered in a non-invasive manner and with a sufficient level of challenge, as sports or an art therapy intervention.

Recent systematic reviews with meta-analyses pointed out that people with severe psychosocial disabilities show lower levels of physical activity [37, 39, 40], higher levels of sedentary behaviors [32] and that interventions based on moderate-vigorous levels of physical activity with the supervision of qualify personnel are needed among this population, to enhance their health and wellbeing [38, 57].

It is known that physical activity stimulates the release of endorphins creating a mild sense of euphoria, and also that synchronized training in a college rowing crew creates a heightened endorphin surge compared with a similar training regime carried out alone. These evidences may explain the sense of euphoria experienced during other social activities that are involved in humans’ social bonding [58, 59].

We advance that the rehabilitative intervention focused on sailing in a crew proposed by “VelaMente?!” project produce an improvement in self-efficacy because it possesses these features: 1) it was focused on a challenging and structured course on sailing; 2) it was a group intervention; 3) it was tailored in naturalistic outdoor place, out of, and relative far from, the mental health services where more traditional rehabilitative activities are usually proposed; 4) the main role of mental health operators was to motivate attenders, facilitate the activities between the participants and the environment (*i.e.*: communication with skippers and sailing teachers; exploring harbor, knowledge about sailboats equipment and tools, *etc*…), promoting both a sense of belonging and a supportive and stimulating climate.

Recent studies about the effectiveness of innovative group psychosocial rehabilitative interventions focused on sport activities and counseling [60], sport activity, mindfulness, psychoeducation [61] and job training [62], even when did not directly considered self-efficacy as an outcome nor mediator on outcomes, seem to provide the opportunity to enhance self-efficacy, self-esteem, self-confidence, and a positive self-image in people with severe psychosocial disabilities [3].

As self-efficacy [12], SoC is a relative stable disposition of personality that is developed during childhood, adolescence, and through adulthood [63, 64] and they are both related with [23], and impact [22] the perceived QoL.

In a randomized-controlled trial [25] about effectiveness of a lifestyle intervention on symptoms, QoL and SoC of people with a psychosocial disability, it was found that SoC significantly improved after a 12-month health intervention in the form of study circles with diet sessions and physical activities with a sufficient level of challenge that encouraged participation in a social context.

In our study, the perceived QoL and SoC tended to improve in the sailing course group during the intervention, even not in a statistically significant way, when compared to the wait list group, in which subjects tended to maintain lower levels on these outcomes with a relative stability during time. Furthermore, improving trend about QoL seemed to partially confirm findings of a previous study [39], in which it was observed a significant improvement on QoL after a six months psychosocial rehabilitative intervention focused on sailing. It could be possible that, to observe significant improvement on QoL, as well as on SoC, requires a more lasting period of treatment.

### 4.1 Limitations

This study presents some limitations. One of these was the small sample size, even if partially moderated by the parallel groups design, with one group crossed to the experimental intervention. It has some advantages against a longitudinal model without this crossing: many subject serves as their own control, hence the effects of confounding factors are restrained. However, the small sample size did not allow to perform multivariate analyses.

Another limit is the relatively high attrition rate, mainly due to the end of the rehabilitation programme in the mental health services regarding an high number of subjects involved in the first group (Group A) attended the sailing course. The dismission of these subjects from their mental health service was the reason why it was not possible to assess the Group A three months after the post-treatment assessment (T2). The lack of follow-up measures did not allow to provide any information on longevity of outcomes and lasting effects of interventions and to perform a cross-over design.

Finally, the use of control interventions that are not evidence based may have determined some bias in favour of the experimental intervention.

## CONCLUSION

The aims of mental health care of people with severe psychosocial disabilities should not be limited to aspects of clinical recovery, like symptoms control and functioning [65]. Care should also aim to increase people’s abilities for a comprehensive social inclusion and to promote their health and well-being within the limitations caused by symptoms, as it is captured in the personal recovery concept [66].

The findings of the present study pointed out that, when compared to more traditional group psychosocial rehabilitative activities, an intervention focused on sailing in a crew significantly promotes self-efficacy in people with severe psychosocial disabilities, and this might enhance the SoC and the QoL in the long term.

Further longitudinal research is needed, also to better evaluate improvements on QoL and SoC. However, shortages in resources in the Sardinian public sector of mental health promotion [67] could not easily allow the development and implementation of psychosocial rehabilitative programs such as “VelaMente?!”, even if it respects many features of new ecological models in health promotion regarding physical activity interventions for people with psychosocial disabilities [68-71].

## Figures and Tables

**Table 1:** Socio-demographic, clinical and psychosocial characteristics of the samples at T0.

**VARIABLES**	**DESCRIPTIVE STATISTICS**		**HOMOGENEITY STATISTICS**
	**SAILING** **COURSE** **(N 33)**	**USUAL REHAB** **TREATMENTS** **(N 18)**	
Socio-demographic			
**Gender** **M** **F**	27 (81.8%) 6 (12.2%)	13 (72.2%) 5 (27.8%)	Chi-Square = 0.194, df =1, p = 0.6599
**Age**	37.76±11.6	36.22±9.17	F = 0.236, df = 1;49, p = 0.629
**Education (years)**	9.3±2.7	9.2±3	F = 0.015, df = 1;49, p = 0.904
**Marital status**	5 (15.2%) 28 (84.8%)	1 (5.6%) 17 (94.4%)	Chi-Square = 0.316, df =1, p=0.5743
**(married)** **yes** **no**			
Clinical			
**Diagnosis**	3 (9.1%) 20 (60.6%) 10 (30.3%)	3 (16.7%) 10 (55.6%) 5 (27.7%)	Chi-Square = 0.6439, df =1, p=0.7247
**Schizophreniaspectrum psychoses** **Affective psychoses** **Personality disorders**			
Psychosocial			
**Sense of coherence (SOC)**	52,15±8,804	53,50±14,085	F = 0.178, df = 1;49, p = 0.675
**Self-efficacy (GSEs)**	28,58±4,981	28,17±4,260	F = 0.087, df = 1;49, p = 0.769
**Quality of Life (SF-12)**	34,48±5,292	33,72±7,984	F = 0.166, df = 1;49, p = 0.685

**Table 2:** Changes over time in measures of sense of coherence, self-efficacy and quality of life.

	**MEAN±SD DIFFERENCES** **from pre- to post-treatment**	**CHANGES** **OVER TIME**	**BETWEEN GROUP** **DIFFERENCES** **OVER TIME**
**SOC**	SC (N 33) pre: 52.15 ± 8,804; post: 54.36 ± 8,317 rTAU (N 18) pre: 53.50± 14.085; post: 53.22 ± 13.122 F = 0.001; df = 1,49; p = 0.971	F = 0.522; df = 1,49; p = 0.473	F = 0.866; df = 1,49; p = 0.357
**GSEs**	SC (N 33) pre: 28.58 ± 4.981; post: 29.67 ± 4.748 rTAU (N 18) pre: 28.17 ± 4.260; post: 26.61 ± 4.865 F = 1.789; df = 1,49; p = 0.187	F = 0.198; df = 1,49; p = 0.659	F = 6.414; df = 1,49; p = 0.015
**SF-12**	SC (N 33) pre: 34.48 ± 5.292; post: 35.67± 5.951 rTAU (N 18) pre: 33.72 ± 7.984; post: 34.17± 7.725 F = 1.165; df = 1,49; p = 0.286	F = 1.165; df = 1,49; p = 0.286	F = 0.240; df = 1,49; p = 0.627

Legend: SC= sailing course; rTAU= rehabilitative treatments as usual.
